# Associations of serum vitamin B12 and its biomarkers with musculoskeletal health in middle-aged and older adults

**DOI:** 10.3389/fendo.2024.1387035

**Published:** 2024-05-14

**Authors:** Jiao Zhao, Qi Lu, Xianfeng Zhang

**Affiliations:** ^1^ Zhejiang University School of Medicine, Hangzhou, Zhejiang, China; ^2^ Shanghai Clinical Research Center of Bone Disease, Department of Osteoporosis and Bone Disease, Shanghai Jiaotong University Affiliated Sixth People’s Hospital, Shanghai, China; ^3^ Department of Endocrinology, Affiliated Hangzhou First People’s Hospital, School of Medicine, Westlake University, Hangzhou, Zhejiang, China

**Keywords:** bone mineral density, osteoporosis, sarcopenia, vitamin B12, hyperhomocysteinemia

## Abstract

**Introduction:**

The effects of vitamin B12 metabolism on musculoskeletal health and the exact mechanism have not been fully determined. Our study aimed to assess the association of vitamin B12 and its biomarkers with musculoskeletal health in middle-aged and older adults.

**Methods:**

The data from the National Health and Nutrition Examination Survey 2001–2002 were used to investigate the effects of serum vitamin B12 and its biomarkers (homocysteine and methylmalonic acid) on skeletal muscle health. Bone mineral density (BMD), lean mass, gait speed and knee extensor strength were used as indicators for musculoskeletal health.

**Results:**

Serum vitamin B12 level was positively correlated with the total and appendicular lean mass (β = 584.83, *P* = 0.044; β = 291.65, *P* = 0.043) in older adults over 65 years of age. In the full population, plasma homocysteine was associated with total lean mass, appendicular lean mass, gait speed, and knee extensor strength (all *P* < 0.05). Among older adults over 65 years of age, homocysteine level was significantly negatively correlated with gait speed and knee extensor strength (β = -12.75, *P* = 0.019; β = -0.06, *P <*0.001). Plasma methylmalonic acid was negatively associated with total BMD and femur BMD in the full population (β = -0.01, *P* = 0.018; β = -0.01, *P* = 0.004). In older adults, methylmalonic acid significantly affected total BMD, femur BMD and knee extensor strength (β = -0.01, *P* = 0.048; β = -0.01, *P* = 0.025; β = -7.53, *P* = 0.015).

**Conclusions:**

Vitamin B12 and its biomarkers are closely related to BMD, body composition, muscle strength and physical function in middle-aged and older adults. Vitamin B12 may be an important indicator of musculoskeletal health in the elderly.

## 1 Introduction

**Figure 1:**
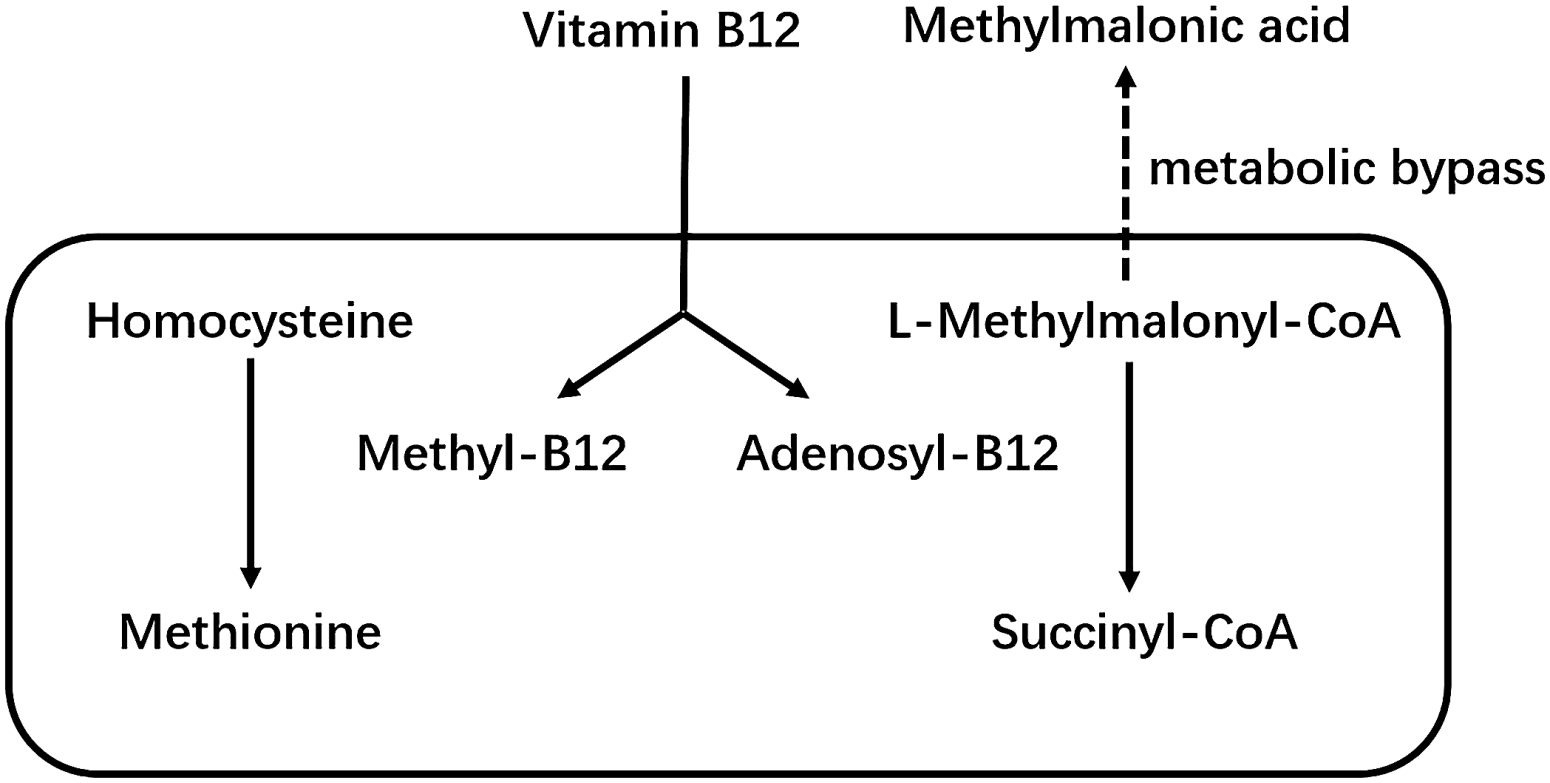
Metabolic pathway of vitamin B12, homocysteine and methylmalonic acid.

Vitamin B12 as a member of the B vitamins, is a hydrosoluble vitamin. It cannot be synthesized by the human body and must be taken up in food or synthesized by the gut microbiota (1). Vitamin B12 is an important cofactor of methionine synthase and L-methylmalonyl-CoA mutase, which are involved in homocysteine and methylmalonic acid metabolism (MMA) (2). **Figure 1** shows the metabolic pathways of vitamin B12, homocysteine and MMA. Low levels of vitamin B12 are associated with homocysteine and MMA. Therefore, these two factors are considered as two functional biomarkers of vitamin B12 deficiency (3). Vitamin B12 deficiency is a fairly common public health problem, occurring mainly in pregnant women and the elderly (4). In addition, recent studies have suggested that vitamin B12 deficiency is associated with a variety of chronic diseases, especially stroke (5), osteoporosis (6), cognitive impairment (7) and physical dysfunction (8, 9). That is why there has been interest in the effects of vitamin B12 on musculoskeletal health. Although studies have shown that vitamin B12 is closely associated with the musculoskeletal system (10), however, the effects of vitamin B12 metabolism on musculoskeletal health and the precise mechanisms remain unclear (11, 12).

Clarke (13) et al. investigated the relationship between B vitamins and bone mineral density (BMD) in patients with celiac disease aged 20 years and older. The study showed a significant association between serum vitamin B12 and BMD in the hip and femur neck, but this protective effect on bone was found only in men (13). In a study of postmenopausal women by Bozkurt (14) et al., low BMD in the femur neck and vertebrae were associated with a significant reduction in serum vitamin B12 levels. In addition, women with osteoporosis had higher levels of homocysteine compared with healthy or osteopenia women (14).The Framingham Study showed that vitamin B12 levels were inversely associated with hip fracture risk, but after controlling for covariates such as BMD, vitamin D and homocysteine levels in a multivariate analysis, this negative correlation was attenuated (15). An analysis by Morris et al. (16) on 1550 participants showed a positive association between serum vitamin B12 concentrations and hip BMD, and this relationship was a dose-response fashion. At the same time, participants with hyperhomocysteinemia had lower BMD than those with normal homocysteine levels (16). According to the Hordaland Homocysteine Study, high homocysteine levels in middle-aged and elderly women were associated with decreased BMD, whereas this association did not exist in men (17). A meta-analysis revealed a 4% reduction in fracture risk for every 50 pmol/L increase in vitamin B12 concentration, whereas a 4% increase in fracture risk for every 1 μmol/L increase in homocysteine concentration (18). A study by Herrmann et al. has shown that B-vitamin supplementation in osteoporosis patients with high homocysteine levels, leading to an increase in lumbar spine BMD and a decrease in bone turnover markers (19). However, Rumbak et al. noted that in healthy women aged 45–65 years, neither vitamin B12, folate, nor homocysteine levels were significantly associated with BMD (20). Furthermore, Holstein et al. found no association between serum levels of vitamin B12 and trabecular thickness, thus questioning the true role of vitamin B12 in bone turnover (21). In a study by Bailey et al., serum vitamin B12 levels were not directly associated with BMD, but their main functional markers, MMA and homocysteine levels, were significantly associated with the risk of developing osteoporosis (22). A randomized controlled trial by Green et al. showed that vitamin B12 supplementation had no effect on bone turnover in healthy older adults (23). Analysis of the HOPE-2 Trial also showed that vitamin B12 supplementation did not reduce fracture risk in a high-cardiovascular-risk population (24).

Many studies have reported the effect of vitamin B12 deficiency on many geriatric syndromes, but to date, only a few cross-sectional studies have investigated the association of vitamin B12 with sarcopenia (25). The study by Mithal et al. suggested that vitamin B12 deficiency may impair muscle function in a homocysteine dependent manner, manifested in reduced gait speed and muscle strength (8). A cross-sectional study by Matteini et al. showed that decreased serum vitamin B12 concentration, increased in MMA concentration, and increased in homocysteine concentration all resulted in decreased muscle mass and impaired physical function in subjects (26). In addition, studies have shown that increased homocysteine concentrations were associated with decreased quadriceps strength and gait speed (27). In contrast, Swart et al. showed that hyperhomocysteinemia was not associated with muscle mass (28). Consistently, no association was observed between low vitamin B12 levels or high homocysteine levels and recurrent falls in the LASA study (6). In a randomized controlled trial study of stroke patients, vitamin B12 was found to have no statistically significant effect on fall rates (29).

At present, studies on the relationship between vitamin B12 and its metabolic markers and musculoskeletal health are still insufficient, and further large cohort studies are needed. Assessment of musculoskeletal system health should include multiple dimensions such as BMD, muscle mass, and function (30). In view of this, this study used data from the National Health and Nutrition Examination Survey (NHANES) to explore the relationship between serum vitamin B12 and its biomarkers (homocysteine and MMA) with BMD, body composition, muscle strength and physical function.

## 2 Materials and methods

### 2.1 Population

The NHANES is a cross-sectional study sponsored annually by the National Center for Health Statistics at the Centers for Disease Control and Prevention (CDC). The study used a nationally representative sample to assess the health and nutritional status of the U.S. civilian population. The NHANES data is released to the public every 2 years and includes standardized physical exams and health-related interviews. All NHANES surveys collect data through household interviews and direct standardized medical examinations at specially equipped mobile examination centers. Details of investigation and the NHANES methodology used can be found in the CDC website (http://www.cdc.gov/nchs/nhanes.htm). The NHANES study is in line with the Declaration of Helsinki and the protocols were approved by the National Center for Health Statistics Ethics Review Board.

We studied individuals enrolled in the NHANES 2001–2002. Vitamin B12 and its metabolic markers, parameters of the musculoskeletal health, along with other information such as demographic, lifestyle, and laboratory test data were measured and recorded. The NHANES 2001–2002 included a total of 11,039 participants, of whom 8390 had their serum vitamin B12 concentrations measured. The NHANES exclusion criteria for dual-energy X-ray absorptiometry (DXA) scans include weight over 136 kg or height over 196 cm, pregnancy, nuclear medicine studies in the past 3 days or a history of contrast agent use (barium) in the past 7 days (31). As a result, a total of 7,126 participants underwent DXA scans. It is important to note that NHANES 2001–2002 only assessed muscle strength and physical function in participants who were older than 50 years old, so only this subset of participants was included in this study. In addition, participants who had a history of brain aneurysm or stroke, severe back pain, a history of myocardial infarction in the past 6 weeks, knee surgery or knee replacement, or chest or abdominal surgery in the past 3 weeks were unable to undergo strength testing and were excluded from this study (32). In the end, out of 7,126 participants, we included only 1,466 participants who completed DXA scans (BMD and body composition), as well as muscle strength and physical function tests. Given that musculoskeletal health is strongly associated with age, we also performed a subgroup analysis of participants over 65 years of age.

### 2.2 Measurement of serum vitamin B12 and its biomarkers

Serum vitamin B12 concentration was measured using Bio-Rad Laboratories “Quantaphase II Folate/vitamin B12” radioassay kit (Bio-Rad Laboratories, 1993) (33). Serum samples were determined in combination with ^57^Co-vitamin B12 in a solution containing dithiothreitol (DTT) and cyanide (33). More detailed information about vitamin B12 concentration measurement can be found on the NHANES website (33). For NHANES 2001, the levels of plasma total homocysteine were determined using the “Abbott Homocysteine IMX (HCY) assay” (34). For NHANES 2002, the plasma total homocysteine concentration was determined by the “Abbott Axsym system” (34). Both methods use fully automated fluorescence polarization immunoassay (FPIA) (34). The FPIA method has been shown to have excellent accuracy (coefficient of variation ≤5%) compared to high-performance liquid chromatography (35). More detailed information on the measurement of plasma total homocysteine levels can be found on the NHANES website (34). MMA was extracted from plasma using strong anion exchange resin and added to internal standard (36). The extracted acid was subsequently derived from cyclohexanol to form dicyclohexyl ester (36). The derivatization samples were injected into gas chromatography to separate the other components (36). The mass selective detector using selected ion monitoring was used to monitor the effluent of the gas chromatograph (36). Results were quantified by using the peak area ratios of MMA and internal calibration of internal standards (36). More detailed information on MMA concentration measurement can be found on the NHANES website (36).

### 2.3 BMD and body composition

DXA scanning was performed on the QDR 4500A fan beam densitometer (Hologic, Inc., Bedford, MA) in accordance with the manufacturer’s guidelines (31). Participants were asked to remove any jewelry or metal objects that might affect the scan results and change into gowns (31). We used available data on total and regional BMD (g/cm^2^), fat mass (g), total and appendicular lean mass (g), and percent body fat (%). Based on the results of the analysis of QDR 4500A DXA data, NHANES adjusted lean mass and fat mass, reducing lean mass by 5% and increasing fat mass by the equivalent weight (31). Further details of the DXA data acquisition protocol are described on the NHANES website (31).

### 2.4 Muscle strength and physical function

The muscle strength and physical function tests were done on participants who were middle−aged and older adults (≥50 years). The average peak force of the knee extensor muscle was measured using the Kin Com Isokinetic Dynamometer (Chattanooga group, Inc., Chattanooga, TN), which required multiple tests (32) (37). Participants did not have to exert their best efforts in the first 3 tests, which were used to learn movements and warm up (32). In the latter 3 trials, muscle strength was measured with maximum effort (32). If the participant completed 4–6 trials, the maximum peak force value was selected. When fewer than 4 trials were completed, the highest values of the remaining trials were used for analysis. The final test result was the peak force (Newton) of the quadriceps at one speed (60 degrees/second) (32). The gait speed test required participants to complete a timed 20 foot (6.1 m) walk at a normal pace (32). Participants could use canes or walkers if needed. The gait speed was then converted to meters per second (m/s) and used for our analysis. The full information for these procedures is available on the NHANES website (32).

### 2.5 Covariates

The demographic and lifestyle characteristics of the participants were collected using a standardized questionnaire during the interview. Information on alcohol consumption, weight and height was obtained during physical examinations at a mobile examination center. Races was categorized as “Mexican American”, “Other Hispanic”, “non-Hispanic White”, “non-Hispanic Black”, and “other race” Educational attainment was classified as “less than high school”, “high school graduate/general education development”, and “more than high school”. The family income to poverty ratio (PIR) is based on the ratio of household income to the poverty level set by the U.S. Department of Health and Human Services (37). PIR was classified as 0–1.0, 1.01–4.99 and 5.0, with higher PIR values indicating higher household income. Body mass index (BMI) values were obtained by calculating height and weight data from physical examination. Alcohol intake habits were based on the answer of “In the past 12 months, how often did you drink any type of alcoholic beverage?”, and the participants were sorted into “never”, “1–2 times”, “3–10 times” and “>10 times”. Smoking status was based on the answer of “Have you smoked at least 100 cigarettes in your entire life?” And “Do you now smoke cigarettes?”. Participants were classified as “non-smoker”, “former smoker” and “current smoker”. “Non-smokers” were participants who had never smoked 100 cigarettes in their lives. “Former smokers” were defined as people who have smoked at least 100 cigarettes in their lifetime, but not currently smoking. “Current smokers” were defined as participants who had smoked at least 100 cigarettes in their lifetime and were still smoking. Physical activity was categorized on the answer of “Moderate activity over past 30 days?” Histories of hypertension, diabetes, renal impairment, liver impairment and cancer were defined as “yes” to the following questions: “Have you ever been told by a doctor or other health professional that you had hypertension, also called high blood pressure?”, “Have you ever been told by a doctor or other health professional that you had diabetes or sugar diabetes?”, “Have you ever been told by a doctor or other health professional that you had weak or failing kidneys?”, “Has a doctor or other health professional ever told you that you had any kind of liver condition?” And “Have you ever been told by a doctor or other health professional that you had cancer or a malignancy of any kind?” Cardiovascular disease (CVD) is based on the answer of these questions: “Has a doctor or other health professional ever told you that you had congestive heart failure?”, “Has a doctor or other health professional ever told you that you had coronary heart disease?”, “Has a doctor or other health professional ever told you that you had angina, also called angina pectoris?” and “Has a doctor or other health professional ever told you that you had a heart attack (also called myocardial infarction)?” If the answer to any of these questions is “yes,” the participant was judged as had CVD. Total vitamin B12 intake and vitamin K intake (μg/day) was measured through in-person dietary intake interviews conducted by trained visitors for the first 24 and 48 hours, and then averaged as final inclusion data. The family history of osteoporosis was based on the answer of “Including living and deceased, were any of your biological that is, blood relatives including grandparents, parents, brothers, sisters ever told by a health professional that they had osteoporosis or brittle bones?”. Total serum calcium was determined using o-cresolphthalein complexone, which reacted with calcium in the presence of 8-hydroxyquinoline to form chromophore. The intensity of the color reaction was proportional to the amount of calcium in the sample. Serum 25-hydroxyvitamin D [25(OH) D] data from NHANES 2001–2002 have been converted by using regression to equivalent 25(OH) D measurements from standardized liquid chromatography-tandem mass spectrometry methods, and used for subsequent analysis. Serum folate concentration was measured using Bio-Rad Laboratories “Quantaphase II Folate/vitamin B12” radioassay kit (Bio-Rad Laboratories, 1993), same as serum vitamin B12. Details of Demographics Data (38), Dietary Data (39), Laboratory Data (40) and Questionnaire Data (41) can be obtained from the NHANES website.

### 2.6 Statistical methods

According to the analysis guidelines developed by NHANES (42), all analyses include sample weights to account for the complex survey design. All analyses were performed using R software (version 4.0.3, available at https://www.R-project.org). *P* value < 0.05 was considered statistically significant. Categorical variables are presented as frequency [percent (%)], while continuous variables are presented as median [interquartile range (IQR)]. Serum vitamin B12 and its biomarkers (homocysteine and MMA) were used as exposure. The musculoskeletal health was assessed using the following parameters: total BMD (g/cm^2^), lumbar spine BMD (g/cm^2^), femoral BMD (g/cm^2^), total lean mass (g), appendicular lean mass (g), gait speed (m/s), and knee extensor strength (Newton). After evaluating the visual inspection of residuals of these data, we found that the linear regression fit was better when these exposures were logarithmically transformed. In the multivariate linear regression analysis, we established three models. Model 1 was an unadjusted model, and Model 2 was adjusted for age and sex. Model 3 was a fully adjusted model using variables selected according to the current literature. These variables included age, sex, race, alcohol consumption, smoking status, height, fat mass (%), physical activity, CVD, diabetes, cancer, liver impairment, kidney impairment, serum folate, serum 25(OH) D, total serum calcium and vitamin K intake. In 1999–2004, approximately 21% of NHANES participants eligible for DXA screening had all or part of their DXA data missing (43). The amount of missing DXA data is larger than other data files, and the missing data appear to have a systematic, non-random pattern. Therefore, the missing DXA data were imputed using the sequential regression imputation method, and a further description can be found in the NHANES document (43). All analyses were repeated in the full population and in a subgroup of older adults (≥65 years).

## 3 Results

### 3.1 Participant characteristics

The median age of participants was 64 (IQR: 56–73) years, with an equal distribution between males and females (50.48% and 49.52%, respectively). **Table 1** shows the characteristics of participants in the full population (≥50 years of age) and the subgroup of older adults (≥65 years of age). The median serum vitamin B12 concentration was 352.76(263.65–467.70) pmol/L in the full population, and 353.13(260.51–471.95) pmol/L in the older adults subgroup. There was no significant difference in vitamin B12 and MMA concentrations between the subgroup and the full population (*P* values were 0.230 and 0.365, respectively), but the homocysteine levels in the elderly individuals were significantly higher than that in the full population (*P* < 0.001)). Except lumbar spine BMD, the total BMD, femur BMD, total lean mass, appendicular lean mass, gait speed and knee extensor strength of the elderly were lower than those of the full population (*P* < 0.001).

**Table 1:** Participant characteristics in the full population (≥ 50 years) and older adults (≥ 65 years).

Variable	Full population (≥ 50 years)	Older adults (≥ 65 years)	*P* value
Vitamin B12 (pmol/L)	352.76 (263.65–467.70)	353.13 (260.51–471.95)	0.230
Homocysteine (μmol/L)	9.15 (7.59–11.26)	9.84 (8.17–12.15)	**<0.001**
Methylmalonic acid (μmol/L)	0.14 (0.11–0.19)	0.16 (0.12–0.21)	0.365
Total BMD (g/cm2)	1.09 (1.00–1.19)	1.05 (0.95–1.16)	**<0.001**
Lumbar Spine BMD (g/cm2)	0.99 (0.88–1.12)	0.97 (0.85–1.12)	0.142
Femur BMD (g/cm2)	1.14 (1.02–1.26)	1.10 (0.97–1.24)	**<0.001**
Total lean mass (g)	47600 (39163–57532)	45134 (37817–53979)	**<0.001**
Appendicular lean mass (g)	20366 (16115–24968)	19094 (15377–23236)	**<0.001**
Knee extensor strength (N)	253.6 (196.8–318.6)	224.6 (175.6–282.6)	**<0.001**
Gait speed (m/s)	1.02 (0.87–1.18)	0.96 (0.80–1.10)	**<0.001**

BMD, bone mineral density. P values were obtained by inter group comparison between the fall population and the older adults subgroup. Bolded values statistically significant.

### 3.2 Associations between the serum vitamin B12 concentration and potential confounders

**Supplementary Table 1** shows a univariate analysis of the association between serum vitamin B12 concentration and potential confounders (demographic, lifestyle, and clinical factors) in the full population. As expected, vitamin B12 levels were inversely correlated with the levels of homocysteine and MMA. Serum vitamin B12 concentration was associated with sex, race, height, weight, BMI, smoking status, vitamin B12 intake, total serum calcium, serum 25(OH)D, total fat mass, as well as certain diseases. Serum vitamin B12 concentrations were negatively correlated with height, weight, and BMI, and positively correlated with vitamin B12 intake, total serum calcium, serum 25(OH)D and total fat mass. In addition, vitamin B12 concentrations were higher in women, Mexican Americans, and non-smokers, and lower in participants with cancer and CVD.

### 3.3 Associations between the serum vitamin B12 concentration and outcome measures

Serum vitamin B12 concentration was associated with lumbar spine BMD, femur BMD, total lean mass, appendicular lean mass, and knee extensor strength (all *P* values < 0.05; **Table 2**). After adjusting for age and sex, serum vitamin B12 concentration was only associated with total lean mass and appendicular lean mass. According to the fully adjusted model, there was no significant correlation between serum vitamin B12 concentration and outcomes in the full population. In the subgroup analysis of older adults over 65 years, the association between serum vitamin B12 and total lean mass, appendicular lean mass remained in the fully adjusted model (β = 584.83, 95%CI 14.84, 1154.73, *P* = 0.044; β = 291.65,95% CI 8.68,574.60, *P* = 0.043) (**Table 3**). As for bone outcomes, there was no significant association between serum vitamin B12 concentration and total BMD, lumbar spine BMD, or femur BMD in the fully adjusted model.

**Table 2:** Multivariate associations between vitamin B12 concentrations and outcome measures in the full population (≥ 50 years).

Variable	Model 1		Model 2		Model 3	
	β [95% CI]	*P* value	β [95% CI]	*P* value	β [95% CI]	*P* value
Total BMD (g/cm2)	0.01 [-0.00, 0.02]	0.115	0.00 [-0.01, 0.01]	0.659	0.00 [-0.01, 0.01]	0.705
Lumbar Spine BMD (g/cm2)	0.02 [0.01, 0.03]	**0.009**	0.01 [-0.00, 0.03]	0.091	0.01 [-0.01, 0.02]	0.210
Femur BMD (g/cm2)	0.02 [0.00, 0.03]	**0.014**	0.00 [-0.01, 0.01]	0.866	0.00 [-0.01, 0.01]	0.668
Total lean mass (g)	1957.45 [1142.74,2771.75]	**<0.001**	788.89 [264.61,1313.16]	**0.003**	229.03 [-193.06,651.15]	0.285
Appendicular lean mass (g)	889.45 [483.92, 1294.97]	**<0.001**	320.98 [57.76, 584.21]	**0.017**	98.02 [-118.78,314.79]	0.376
Knee extensor strength (N)	6.51 [0.05, 12.98]	**0.048**	1.15 [-3.98, 6.28]	0.660	2.00 [-3.36, 7.37]	0.468
Gait speed (m/s)	0.00 [-0.02, 0.02]	0.785	0.00 [-0.01, 0.02]	0.776	-0.00 [-0.02, 0.02]	0.912

BMD, bone mineral density. The coefficient (β) with 95% CI represents the percentage difference in the variable as vitamin B12 increases for 1-fold. Model 1, unadjusted; Model 2, adjusted for age and sex. Model 3, age, sex, race, smoking status, alcohol intake, height, fat mass (%), physical activity, cardiovascular disease, diabetes, cancer, liver impairment, renal impairment, serum folate, serum 25(OH) D, total serum calcium and vitamin K intake. All regressions were also accounted for complex survey design using the sampling weights provided. Bolded values statistically significant.

**Table 3:** Multivariate associations between vitamin B12 concentrations and outcome measures in older adults (≥ 65 years).

Variable	Model 1		Model 2		Model 3	
	β [95% CI]	*P* value	β [95% CI]	*P* value	β [95% CI]	*P* value
Total BMD (g/cm2)	-0.01 [-0.03, 0.00]	0.153	0.01 [-0.00, 0.02]	0.167	0.01 [-0.01, 0.02]	0.293
Lumbar Spine BMD (g/cm2)	-0.02 [-0.04, 0.00]	0.0616	-0.00 [-0.02, 0.02]	0.753	0.01 [-0.02, 0.03]	0.603
Femur BMD (g/cm2)	-0.02 [-0.04, -0.00]	**0.0197**	0.01 [-0.01, 0.02]	0.344	0.01 [-0.01, 0.02]	0.348
Total lean mass (g)	2459.49 [1391.87, 3527.08]	**<0.001**	832.94 [137.78, 1528.10]	**0.019**	584.83 [14.84, 1154.73]	**0.044**
Appendicular lean mass (g)	1111.74 [588.49, 1634.97]	**<0.001**	331.48 [-13.07, 676.04]	**0.050**	291.65 [8.68, 574.60]	**0.043**
Knee extensor strength (N)	9.17 [1.25, 17.09]	**0.023**	2.89 [-3.75, 9.53]	0.393	5.90 [-1.01, 12.85]	0.089
Gait speed (m/s)	0.00 [-0.02, 0.03]	0.813	-0.00 [-0.03, 0.02]	0.839	0.01 [-0.01, 0.03]	0.380

BMD, bone mineral density. The coefficient (β) with 95% CI represents the percentage difference in the variable as vitamin B12 increases for 1-fold. Model 1, unadjusted; Model 2, adjusted for age and sex; Model 3, age, sex, race, smoking status, alcohol intake, height, fat mass (%), physical activity, cardiovascular disease, diabetes, cancer, liver impairment, renal impairment, serum folate, serum 25(OH) D, total serum calcium and vitamin K intake. All regressions were also accounted for complex survey design using sampling weights provided. Bolded values statistically significant.

### 3.4 Associations between plasma homocysteine levels and outcome measures

In the full population, homocysteine levels were inversely associated with total lean mass, appendicular lean mass, gait speed, and knee extensor strength after full adjustment for covariates (all *P* values < 0.05) (**Table 4**). According to the results of the subgroup analysis of older adults, homocysteine concentrations were associated with gait speed and knee extensor strength in the fully adjusted model (β = -0.06, 95% CI -0.10, -0.03, *P <*0.001; β = -12.75, 95%CI-23.17, -2.35, *P* = 0.019) (**Table 5**). In addition, there were no significant correlations between homocysteine levels and BMD in the full population and subgroup of older adults.

**Table 4:** Multivariate associations between homocysteine levels and outcome measures in the full population (≥ 50 years).

Variable	Model 1		Model 2		Model3	
	β [95% CI]	*P* value	β [95% CI]	*P* value	β [95% CI]	*P* value
Total BMD (g/cm2)	0.01 [-0.01, 0.02]	0.267	0.00 [-0.01, 0.01]	0.920	-0.01 [-0.02, 0.01]	0.435
Lumbar Spine BMD (g/cm2)	0.03 [0.01, 0.05]	**0.002**	0.02 [-0.00, 0.04]	0.071	0.00 [-0.02, 0.03]	0.789
Femur BMD (g/cm2)	0.02 [0.01, 0.04]	**0.010**	-0.00 [-0.02, 0.01]	0.652	-0.01 [-0.03, 0.00]	0.090
Total lean mass (g)	2518.41 [1308.60, 3728.29]	**<0.001**	570.28 [-265.93, 1406.49]	0.181	-860.15 [-1535.85, -184.45]	**0.015**
Appendicular lean mass (g)	1126.36 [524.00, 1728.69]	**<0.001**	281.67 [-137.61, 700.94]	0.188	-442.82 [-789.92, -95.67]	**0.013**
Knee extensor strength (N)	-17.59 [-27.15, -8.04]	**<0.001**	-14.08 [-22.21, -5.95]	**<0.001**	-9.65 [-18.28, -1.03]	**0.028**
Gait speed (m/s)	-0.13 [-0.16, -0.11]	**<0.001**	-0.08 [-0.11, -0.06]	**<0.001**	-0.06 [-0.08, -0.03]	**<0.001**

BMD, bone mineral density. The coefficient (β) with 95% CI represents the percentage difference in the variable as vitamin B12 increases for 1-fold. Model 1, unadjusted; Model 2, adjusted for age and sex; Model 3, age, sex, race, smoking status, alcohol intake, height, fat mass (%), physical activity, cardiovascular disease, diabetes, cancer, liver impairment, renal impairment, serum folate, serum 25 (OH) D, total serum calcium and vitamin K intake. All regressions were also accounted for complex survey design using sampling weights provided. Bolded values statistically significant.

**Table 5:** Multivariate associations between homocysteine levels and outcome measures in older adults (≥ 65 years).

Variable	Model 1		Model 2		Model3	
	β [95% CI]	*P* value	β [95% CI]	*P* value	β [95% CI]	*P* value
Total BMD (g/cm2)	0.02 [-0.01, 0.04]	0.132	-0.00 [-0.02, 0.01]	0.673	-0.01 [-0.03, 0.01]	0.352
Lumbar Spine BMD (g/cm2)	0.05 [0.02, 0.08]	**0.002**	-0.02 [-0.01, 0.04]	0.319	-0.00 [-0.03, 0.03]	0.996
Femur BMD (g/cm2)	0.03 [-0.00, 0.05]	0.058	-0.01 [-0.03, 0.01]	0.419	-0.02 [-0.04, 0.00]	0.097
Total lean mass (g)	2206.98 [687.32, 3727.32]	**0.004**	534.65 [-490.06, 1559.35]	0.306	-763.95 [-1619.90, 92.12]	0.083
Appendicular lean mass (g)	1016.99 [273.02, 1760.92]	**0.007**	268.45 [-238.31, -775.21]	0.299	-377.65 [-815.07, 59.81]	0.095
Knee extensor strength (N)	-14.34 [-25.52, -3.16]	**0.012**	-13.51 [-23.22, -3.79]	**0.007**	-12.75 [-23.17, -2.35]	**0.019**
Gait speed (m/s)	-0.11 [-0.14, -0.07]	**<0.001**	-0.08 [-0.12, -0.05]	**<0.001**	-0.06 [-0.10, -0.03]	**<0.001**

BMD, bone mineral density. The coefficient (β) with 95% CI represents the percentage difference in the variable as vitamin B12 increases for 1-fold. Model 1, unadjusted; Model 2, adjusted for age and sex; Model 3, age, sex, race, smoking status, alcohol intake, height, fat mass (%), physical activity, cardiovascular disease, diabetes, cancer, liver impairment, renal impairment, serum folate, serum 25 (OH) D, total serum calcium and vitamin K intake. All regressions were also accounted for complex survey design using sampling weights provided. Bolded values statistically significant.

### 3.5 Associations between the plasma MMA concentration and outcome measures

According to the fully adjusted model, MMA concentration was negatively correlated with total BMD and femur BMD (β = -0.01, 95%CI -0.02, -0.00, *P* = 0.018; β = -0.01, 95%CI -0.02, -0.00, *P* = 0.004) (**Table 6**). The associations between MMA and total BMD, femur BMD and knee extensor strength were maintained in a fully adjusted model of subgroup analysis of older adults (β = -0.01,95% CI -0.02, 0.00, *P* = 0.048; β = -0.01,95% CI -0.03, -0.00, *P* = 0.025; β = -7.53,95% CI-14.10, -0.14, *P* = 0.015) (**Table 7**).

**Table 6:** Multivariate associations between methylmalonic acid levels and outcome measures in the full population (≥ 50 years).

Variable	Model 1		Model 2		Model3	
	β [95% CI]	*P* value	β [95% CI]	*P* value	β [95% CI]	*P* value
Total BMD (g/cm2)	-0.02 [-0.03, -0.01]	**<0.001**	-0.01 [-0.02, -0.00]	**0.008**	-0.01 [-0.02, -0.00]	**0.018**
Lumbar Spine BMD (g/cm2)	-0.00 [-0.02, 0.01]	0.829	0.00 [-0.01, 0.01]	0.972	0.00 [-0.01, 0.02]	0.873
Femur BMD (g/cm2)	-0.02 [-0.03, -0.01]	**<0.001**	-0.01 [-0.02, -0.00]	**0.007**	-0.01 [-0.02, -0.00]	**0.004**
Total lean mass (g)	-674.16 [-1505.59, 157.30]	0.112	8.61 [-549.94, 567.16]	0.976	-197.17 [-646.12, 252.05]	0.392
Appendicular lean mass (g)	-477.86 [-890.89, -64.84]	**0.0234**	-79.78 [-359.84, 200.28]	0.576	-133.35 [-363.85, 97.17]	0.255
Knee extensor strength (N)	-19.49 [-25.97, -13.01]	**<0.001**	-6.07 [-11.50, -0.64]	**0.028**	-4.96 [-10.67, 0.74]	0.095
Gait speed (m/s)	-0.06 [-0.08, -0.05]	**<0.001**	-0.02 [-0.04, -0.00]	**0.019**	-0.02 [-0.03, 0.00]	0.089

BMD, bone mineral density. The coefficient (β) with 95% CI represents the percentage difference in the variable as vitamin B12 increases for 1-fold. Model 1, unadjusted; Model 2, adjusted for age and sex; Model 3, age, sex, race, smoking status, alcohol intake, height, fat mass (%), physical activity, cardiovascular disease, diabetes, cancer, liver impairment, renal impairment, serum folate, serum 25(OH) D, total serum calcium and vitamin K intake. All regressions were also accounted for complex survey design using sampling weights provided. Bolded values statistically significant.

**Table 7:** Multivariate associations between methylmalonic acid levels and outcome measures in older adults (≥ 65 years).

Variable	Model 1		Model 2		Model3	
	β [95% CI]	*P* value	β [95% CI]	*P* value	β [95% CI]	*P* value
Total BMD (g/cm2)	-0.01 [-0.03, 0.00]	0.090	-0.01 [-0.02, 0.00]	0.064	-0.01 [-0.02, 0.00]	**0.048**
Lumbar Spine BMD (g/cm2)	0.01 [-0.01, 0.03]	0.196	0.01 [-0.01, 0.02]	0.617	0.00 [-0.02, 0.02]	0.683
Femur BMD (g/cm2)	-0.01 [-0.03, 0.01]	0.174	-0.01 [-0.02, 0.00]	0.056	-0.01 [-0.03, -0.00]	**0.025**
Total lean mass (g)	-136.20 [-1106.79, 834.34]	0.783	33.43 [-605.30, 672.16]	0.918	-296.73 [-830.35, 237.09]	0.289
Appendicular lean mass (g)	-169.82 [-644.58, 304.89]	0.483	-58.08 [-374.22, 258.07]	0.718	-245.63 [-518.03, 26.85]	0.083
Knee extensor strength (N)	-14.08 [-21.14, -7.01]	**<0.001**	-8.00 [-14.06, -1.95]	**0.010**	-7.53 [-14.10, -0.14]	**0.015**
Gait speed (m/s)	-0.04 [-0.07, -0.02]	**<0.001**	-0.02 [-0.04, -0.00]	**0.042**	-0.02 [-0.04, 0.00]	0.237

BMD, bone mineral density. The coefficient (β) with 95% CI represents the percentage difference in the variable as vitamin B12 increases for 1-fold. Model 1, unadjusted; Model 2, adjusted for age and sex; Model 3, age, sex, race, smoking status, alcohol intake, height, fat mass (%), physical activity, cardiovascular disease, diabetes, cancer, liver impairment, renal impairment, serum folate, serum 25(OH) D, total serum calcium and vitamin K intake. All regressions were also accounted for complex survey design using sampling weights provided. Bolded values statistically significant.

## 4 Discussion

In this study, we found that the association between serum vitamin B12 concentration and musculoskeletal health was only present in older adults. In people over 60 years of age, vitamin B12 was associated with total and appendicular lean mass. Homocysteine levels were inversely associated with muscle strength and physical function, total and appendicular lean mass. A similar association was observed in our subgroup analysis of older adults over 65 years of age. The MMA levels were negatively correlated with total BMD and femur BMD in the full population. In the elderly population, MMA was inversely associated with total BMD, femur BMD and knee extensor strength. Notably, this is the first study to report the relationship between vitamin B12 and its biomarkers and musculoskeletal health in the same cohort.

Ates Bulut et al. conducted a prospective study and found that lean mass, total bone mass and skeletal muscle mass index in the vitamin B12 insufficient group were lower than those in the vitamin B12 sufficient group in the elderly over 60 years of age (44). A study of 2325 adults aged 70–84 years by Chae et al. found that vitamin B12 deficiency was associated with decreased muscle mass, but not with the incidence of sarcopenia and decreased physical function (45). Our study also found an association between B12 deficiency and lower lean mass. However, sarcopenia is characterized not only by low muscle mass, but also by decreased muscle strength and function. Whether there is a direct relationship between vitamin B12 and sarcopenia incidence remains controversial. A case-control study by Verlaan et al. showed that serum vitamin B12 levels were 15% lower in patients with sarcopenia than those in the control group, but the causal relationship remained unclear (25). In a study of 427 hospitalized older adults aged ≥80 years, Tao et al. found that serum vitamin B12 levels had no effect on the incidence of sarcopenia (46). Another study of 731 community-dwelling adults aged ≥65 years investigated the impact of nutritional factors on sarcopenia, and found no correlation between vitamin B12 and muscle mass (47). Singh et al. found that changes in vitamin B12 metabolism generally have little impact on muscle mass and function compared to bone (48). The relationship between vitamin B12 and sarcopenia, including muscle mass and muscle function, is not well understood. Our population-based study provides evidence that vitamin B12 levels affect lean mass. However, further studies are needed to more fully and thoroughly investigate the effects of vitamin B12 on the development of sarcopenia.

Our study found that homocysteine levels were negatively correlated with muscle strength, body function, total lean mass and appendicular lean mass. There are several possible explanations for the differences in muscle strength and physical function associated with hyperhomocysteinemia. Firstly, hyperhomocysteinemia may disrupt the structural integrity of elastin, collagen and proteoglycans, leading to decreased muscle strength (18). In addition, hyperhomocysteinemia may cause angiotoxicity and atherosclerosis by targeting vascular smooth muscle, endothelial cells and thrombocytes, leading to muscle atrophy and decreased muscle strength (27). Secondly, hyperhomocysteinemia can increase the release of reactive oxygen species, which may lead to mitochondrial damage and subsequent inflammation (49). Hyperhomocysteinemia reduces nitric oxide bioavailability and blood flow to muscle cells, which may also contribute to muscle mass (50). In fact, several clinical studies have shown a negative effect of hyperhomocysteinemia on muscle mass and physical function. In the Baltimore Longitudinal Study of Aging, homocysteine levels in healthy women over 50 years of age were negatively correlated with grip strength and gait speed (51). Kirk et al. also reported negative effect of homocysteine on lower limb muscle strength and physical function, and can further cause falls and fractures (52).

Sarcopenia is defined as a geriatric syndrome associated with decreased muscle mass, muscle strength, and/or physical function (53). The main parameters currently available for diagnosis and assessment of sarcopenia are lean mass, muscle strength, and physical function. To date, it has been unclear whether vitamin B12 and its biomarkers are associated with specific components of sarcopenia. Our study suggested that vitamin B12 and its biomarkers were associated with different aspects of sarcopenia, possibly because homocysteine and MMA can affect muscle health in ways independent of vitamin B12. Further studies can use magnetic resonance imaging or high-resolution computed tomography to directly measure the muscle mass/volume to get more accurate conclusions.

In our study, we found that MMA levels were primarily associated with BMD. In the full population and elderly subgroup analysis, we found a negative association between MMA concentration and total BMD and femur BMD. At present, the exact relationship between vitamin B12 metabolism and bone health is not fully established. The results of preclinical studies on the effects of vitamin B12 on bone are also inconsistent. Decreased vitamin B12 concentration and subsequent increased levels of homocysteine and MMA have different effects on multiple intracellular pathways (54). A study by Bailey et al. have shown that vitamin B12 can have an impact on bone health through multiple pathways, including increasing osteoclast formation, altering osteoblast function, and regulating collagen crosslinking (55). Roman-Garcia et al. reported that vitamin B12 deficiency caused stunted growth and decreased bone mass in mice, and that this phenomenon was not due to the accumulation of MMA or homocysteine (54). The authors speculate that it was down-regulated due to the production of other vitamin B12 dependent downstream metabolites (54). Kim et al. used vitamin B12 to stimulate two osteosarcoma cell lines and found functional and dose-dependent proliferative responses, suggesting that vitamin B12 can directly inhibit osteoblast activity (56). In a model of mouse fracture healing, vitamin B12 deficiency, although causing hyperhomocysteinemia, had no effect on fracture repair (57). Singh et al. noted that in mouse models, vitamin B12 deficiency affected parameters including thickness, number and connectivity of trabeculae, as well as cortical thickness and porosity (48). The structural deterioration of cortical and trabecular bone led to a substantial decrease in the density and content of bone minerals in the whole body, further leading to a decline in the biomechanical properties of long bone (48). A Study by Herrmann et al. showed that vitamin B12 deficiency stimulated osteoclasts in a homocysteine-dependent manner, but did not affect osteoblasts (58). However, the authors also found that prolonged vitamin B12 deficiency induced significant homocysteine accumulation in healthy rats, and that these metabolic changes had no adverse effects on bone (58). At present, there is no clear conclusion on the exact relationship between vitamin B12 and bone metabolism, and there are few studies on the relationship between MMA and musculoskeletal health. Our study showed that MMA is inversely associated with total BMD and femur BMD. Some studies have shown that increased MMA concentrations were more strongly associated with poor functional performance than serum vitamin B12 (59). NHANES has also been aware of this problem and, in a roundtable, clarified that MMA concentrations were more reflective of early vitamin B12 status and marginal vitamin B12 deficiency than serum vitamin B12 (60). This could also explain why MMA concentrations were significantly negatively associated with BMD, while vitamin B12 levels were not.

This study has some advantages and limitations. The data for this study came from NHANES, which underwent robust quality assurance and control procedures. The NHANES study included a large representative sample, so there is less potential for sampling bias. In addition, our analysis took into account various factors known to influence vitamin B12 and its metabolic markers and musculoskeletal health. Therefore, our study can better eliminate confounding factors that may appear in multiple regression models. Moreover, this study included 1,466 participants and was more capable of produce meaningful results due to the large sample size provided by NHANES. The main limit of this study is that cross-sectional studies cannot explain causality, and follow-up prospective cohort studies should be conducted to verify these findings. Second, there are residual confounding factors that cannot be eliminated in population-based studies. Finally, current evaluation parameters for musculoskeletal health are still not specific enough, and more accurate measurements of muscle mass/function and bone structure are needed to strengthen our ability to examine the relationship between vitamin B12 and musculoskeletal health.

In this nationally representative sample of middle-aged and older adults, we found that vitamin B12 was associated with total and appendicular lean mass in adults over 60 years of age. Homocysteine levels were inversely associated with muscle strength/physical function, total lean mass, and appendicular lean mass. The MMA concentrations were negatively correlated with total BMD and femur BMD. In general, vitamin B12 and its biomarkers are closely related to musculoskeletal status in middle-aged and elderly people. These three indicators may reflect different changes in BMD, body composition and physical function, respectively, and can be used as important indicators of musculoskeletal health status in elderly people. Further researches are needed to determine the possible mechanisms behind this phenomenon. In addition, more prospective studies are needed to investigate the relationship between vitamin B12 and BMD, muscle strength, and physical function.

## Data availability statement

The original contributions presented in the study are included in the article/**Supplementary Material**. Further inquiries can be directed to the corresponding author.

## Ethics statement

The institutional review board approved the NHANES protocol of the Centers for Disease Control and Prevention (CDC). The studies were conducted in accordance with the local legislation and institutional requirements. The participants provided their written informed consent to participate in this study.

## Author contributions

JZ: Data curation, Investigation, Writing – original draft. QL: Formal analysis, Methodology, Writing – original draft. XZ: Funding acquisition, Supervision, Writing – review & editing.
