# Supplementary material for: Associations of serum vitamin B12 and its biomarkers with musculoskeletal health in middle-aged and older adults
Source: Front Endocrinol (Lausanne). 2024 May 14;15:1387035. doi: 10.3389/fendo.2024.1387035 (PMC11130390; doi:10.3389/fendo.2024.1387035)
Supplement: Supplementary file 1 [file Table_1.docx]

Supplementary Table 1 Participant characteristics and univariate associations between vitamin B12 concentrations and potential confounders in the full population

| Variable | Median (IQR) or n (%) | β[95%CI] | *P* value |
| --- | --- | --- | --- |
| Vitamin B12(pmol/L) | 352.76(263.65-467.70) |  |  |
| Homocysteine (μmol/L) | 9.15(7.59-11.26) | 0.971[0.966, 0.975] | <0.001 |
| Methylmalonic acid (μmol/L) | 0.14(0.11-0.19) | 0.566[0.512, 0.627] | <0.001 |
| Age(years) | 64 (56-73) | 1.000[0.997, 1.002] | 0.770 |
| Sex |  |  |  |
| Men | 740(50.48%) |  |  |
| Women | 726(49.52%) | 1.109[1.054, 1.167] | <0.001 |
| Race |  |  |  |
| Mexican American | 223(15.21%) |  |  |
| Other Hispanic | 43(2.93%) | 0.909[0.773, 1.069] | 0.249 |
| Non-Hispanic White | 934(63.71%) | 0.919[0.855, 0.989] | 0.023 |
| Non-Hispanic Black | 228(15.55%) | 0.993[0.906, 1.089] | 0.887 |
| Other Race | 38(2.59%) | 0.997[0.840, 1.183] | 0.974 |
| PIR |  |  |  |
| 0-1.0 | 154(10.50%) |  |  |
| 1.01-4.99 | 843(57.50%) | 1.029[0.945, 1.121] | 0.511 |
| 5.0 | 361(24.62%) | 1.018[0.926, 1.118] | 0.714 |
| Unknown | 108(7.37%) | 1.130[1.000, 1.277] | 0.050 |
| Education |  |  |  |
| <High school | 432(29.47%) |  |  |
| High school/GED | 324(22.10%) | 1.010[0.940, 1.085] | 0.796 |
| >High school | 709(48.36%) | 1.010[0.951, 1.072] | 0.749 |
| Unknown | 1(0.07%) | 1.551[0.583, 4.124] | 0.379 |
| Weight(kg) | 77.20 (66.10-89.60) | 0.997[0.995, 0.998] | <0.001 |
| Height(cm) | 166.90 (159.60-174.80) | 0.995[0.992, 0.997] | <0.001 |
| BMI(kg/m2) | 27.43 (24.49-30.84) | 0.993[0.988, 0.998] | 0.007 |
| Moderate activity over past 30 days | |  |  |
| Yes | 660(45.02%) |  |  |
| No | 767(52.32%) | 0.951[0.903, 1.002] | 0.059 |
| Unable to do | 38(2.59%) | 0.997[0.847, 1.173] | 0.971 |
| Unknown | 1(0.07%) | 1.500[0.565, 3.984] | 0.415 |
| Smoking status |  |  |  |
| Non-smoker | 667(45.50%) |  |  |
| Former smoker | 569(38.81%) | 0.940[0.889, 0.994] | 0.029 |
| Current smoker | 226(15.42%) | 0.901[0.836, 0.971] | 0.006 |
| Unknown | 4(0.27%) | 0.800[0.491, 1.304] | 0.371 |
| Number of drinking days over last 12 months | |  |  |
| Never | 362(24.69%) |  |  |
| 1-2 times | 399(27.22%) | 0.984[0.917, 1.056] | 0.655 |
| 3-10 times | 403(27.49%) | 0.959[0.893, 1.029] | 0.241 |

Supplementary Table 1 (continued)

| Variable | Median (IQR) or n (%) | β[95%CI] | *P* value |
| --- | --- | --- | --- |
| >10 times | 55(3.75%) | 0.944[0.820, 1.087] | 0.424 |
| Unknown | 247(16.85%) | 1.067[0.984, 1.156] | 0.115 |
| Ever told you had hypertension | |  |  |
| Yes | 635(43.32%) |  |  |
| No | 828(56.48%) | 0.975[0.926, 1.027] | 0.336 |
| Unknown | 3(0.20%) | 1.421[0.808, 2.500] | 0.223 |
| Ever told you had diabetes |  |  |  |
| Yes | 201(13.71%) |  |  |
| No | 1230(83.90%) | 0.953[0.884, 1.026] | 0.200 |
| Unknown | 35(2.39%) | 0.941[0.787, 1.125] | 0.502 |
| Ever told you had renal impairment | |  |  |
| Yes | 39(2.66%) |  |  |
| No | 1420(96.86%) | 0.951[0.811, 1.114] | 0.532 |
| Unknown | 7(0.48%) | 1.158[0.776, 1.729] | 0.473 |
| Ever told you had liver impairment | |  |  |
| Yes | 46(3.14%) |  |  |
| No | 1417(96.66%) | 0.920[0.795, 1.065] | 0.263 |
| Unknown | 3(0.20%) | 1.395[0.780, 2.496] | 0.262 |
| Ever told you had cancer |  |  |  |
| Yes | 227(15.48%) |  |  |
| No | 1238(84.45%) | 1.026[0.956, 1.101] | 0.474 |
| Unknown | 1(0.07%) | 3.873[1.459, 10.286] | 0.007 |
| Ever told you had cardiovascular disease | |  |  |
| Yes | 204(13.92%) |  |  |
| No | 1252(85.40%) | 1.094[1.017, 1.178] | 0.016 |
| Unknown | 10(0.68%) | 1.315[0.959, 1.803] | 0.089 |
| Blood relatives have osteoporosis | |  |  |
| Yes | 195(13.30%) |  |  |
| No | 1214(82.81%) | 0.995[0.923, 1.073] | 0.900 |
| Unknown | 57(3.89%) | 0.984[0.849, 1.139] | 0.825 |
| Serum folate(nmol/L) | 26.7 (19.4-36.7) | 1.003[0.995, 1.010] | 0.057 |
| Vitamin B12 intake(μg/day) | 0 (0-0.13) | 1.033[1.001, 1.066] | 0.042 |
| Vitamin K intake (μg/day) | 65.3(40.2-118.5) | 0.993[0.949, 1.007] | 0.521 |
| Total serum calcium (mmol/L) | 2.35(2.30-2.43) | 1.432[1.112, 1.843] | 0.005 |
| Serum 25(OH)D(nmol/L) | 58.72(44.46-72.97) | 1.005[1.004, 1.006] | <0.001 |
| Total fat mass(g) | 26642(21392-33268) | 1.000[1.000, 1.000] | 0.015 |
| Total percent fat (%) | 35.10(29.23-41.60) | 1.001[0.998, 1.004] | 0.441 |

The coefficient (β*)* with 95% CI represents the fold difference in vitamin B12 concentration as the variable increases for 1 unit.
